# Commentary: “Silver Nanoparticles Coated Poly(L-Lactide) Electrospun Membrane for Implant Associated Infections Prevention”

**DOI:** 10.3389/fphar.2021.759304

**Published:** 2021-10-28

**Authors:** Selvaraj Vimalraj, Saravanan Sekaran

**Affiliations:** ^1^ Centre for Biotechnology, Anna University, Chennai, India; ^2^ Department of Pharmacology, Saveetha Dental College and Hospitals, Saveetha Institute for Medical and Technical Sciences, Chennai, India

**Keywords:** PLLA fiber, guided bone regeneration (GBR), nanoparticle, silver, osteoblasts

## Introduction

Jiaolong et al. (2020) published an interesting study “Silver Nanoparticles Coated Poly(L-Lactide) Electrospun Membrane for Implant-Associated Infections Prevention” on which I would like to express my viewpoints which may benefit other readers and researchers.

The guided bone regeneration (GBR) barrier membrane enhances the bone volume by allowing selective migration of estrogenic cells from boney margins and impeding other cells such as fibroblasts and epithelial cells from overlying mucosa in ridge augmentation procedures (Mellonig and Nevins, 1995). GBR membranes using resorbable and non-resorbable membranes can aid in treating moderate to severe bone defects. An FDA-approved PLLA polymer has been used clinically, and one of the common polymers is used in GBR procedures. Pure PLLA fibers often lack the suitability to support cell adhesion and proliferation diminishing its biological properties (Khatri et al., 2016). In addition to this, polymicrobial biofilm formation on the implanted material surface is the major reason for GBR membrane failure *in vivo* impeding its medical applications (Xie et al., 2010, Arciola et al., 2015, Willems et al., 2016; Trobos et al., 2018; Hong et al., 2019). To circumvent this limitation, the authors have utilized the intrinsic reduction property of polydopamine to ensure *in situ* synthesis of silver nanoparticles on PLLA surface imparting antimicrobial property. This study presents a new method in coating which may be utilized for coating many other nanoparticles and explored for bone tissue engineering applications.

## Harnessing the Property of PDA to Coat Various Metal Nanoparticles on PLLA

**FIGURE 1:**
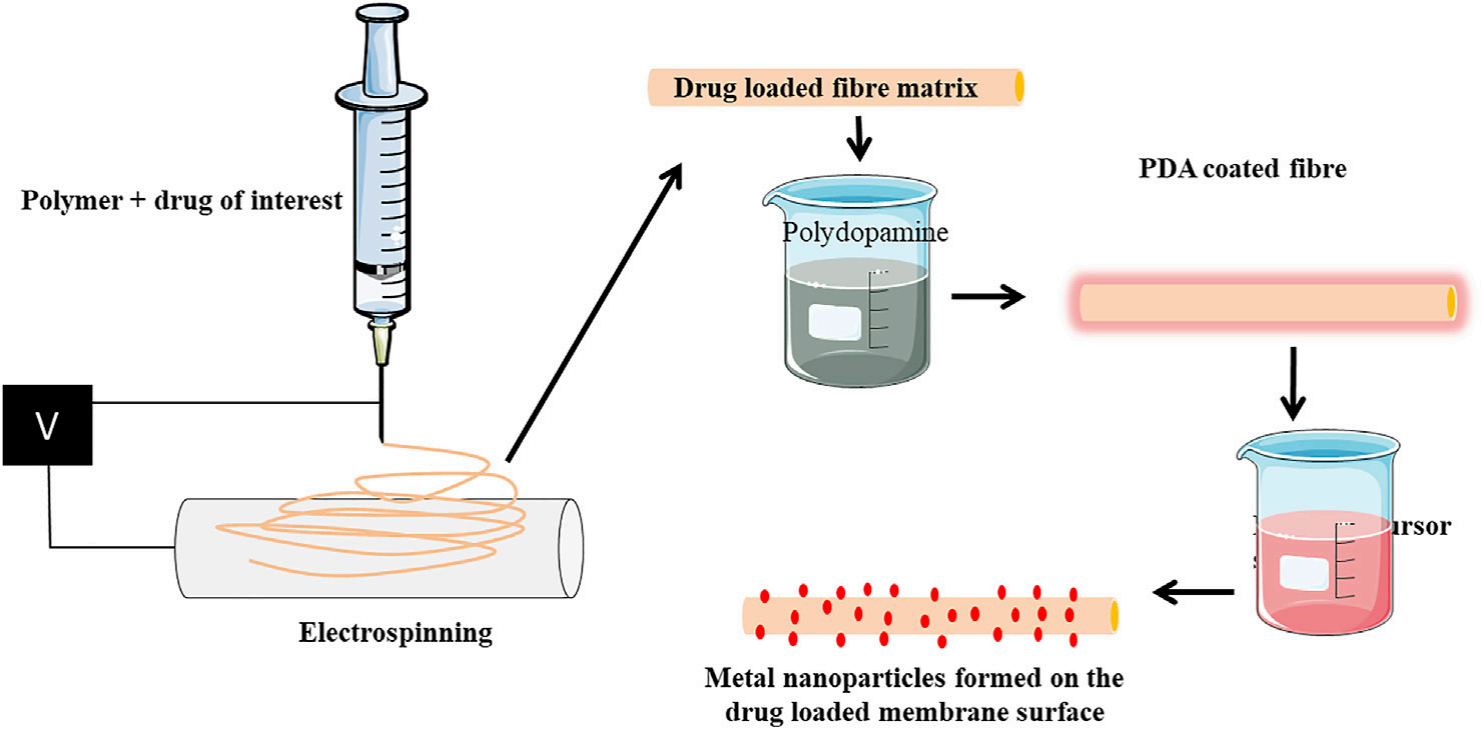
Scheme representing the expansion of the ideology provided by Jialong et al. (2020). Drug can be incorporated into the PLLA fiber by mixing polymer and drug solution prior to electrospinning. The electron spun matrix can be coated with PDA followed by immersion into respective metal precursor solution. The membrane will have drug loaded into them and metallic nanoparticles will be formed on the surface. Such matrix can be used for various tissue engineering purposes for carrying drugs along with metallic nanoparticles.

Polydopamine is an interesting polymer, attributed to the presence of catechol moieties in its building blocks is similar to the mussel foot proteins have gained recent attention for grafting various biological agents (Ryu et al., 2010; Cui et al., 2012 Sun et al., 2014; Ding et al., 2016). Lee et al., 2007, reported self-polymerization of dopamine into thin films on a range of inorganic and organic materials by a simple dip-coating method. For instance, Liu et al., 2019 reported the coating of PLLA nanofiber scaffolds with osteogenic growth peptide *via* PDA coating enhanced cell adhesion, proliferation, osteogenic differentiation, and mineralization. PDA offers a simple, inexpensive, and versatile route compared with conventional surface modification methods in bone tissue engineering (Huang et al., 2016). Jialong utilized the self-polymerization and reduction property of PDA and dip-coated PLLA@PDA membrane in silver nitrate solution to mediate silver nanoparticle formation. This mussel-inspired chemistry of PDA was previously shown to reduce the silver precursor-[Ag(NH3)_2_]^+^ ions to metallic silver nanoparticles, mediated by amine and cathecol groups (Wu et al., 2015). Metal nanoparticles represent an emerging class of materials and known to regulate stem cell differentiation and osteoblast functions favoring bone formation. Gold, silver, iron, nickel, copper, strontium, selenium, and zirconium nanoparticles are known to regulate osteoblast functions (Suh et al., 2013; Zheng et al., 2014; Ye and Shi, 2018; Zhang et al., 2018; Eivazzadeh-Keihan et al., 2020; Tang et al., 2021). In addition to this, metal nanoparticles are known for their excellent antimicrobial properties. Antimicrobial resistance (AMR) is now a global concern halting the use of antibiotics and looking for alternative strategies. Metal nanoparticles offer promising hope in combating AMR. New antimicrobial chemistries are employed enabling the activation of antimicrobial functionality on demand without the need of antibiotics. Antimicrobial photodynamic therapy is of great interest in the recent past and an efficient alternative to the use of antibiotic-based therapies (Cieplik et al., 2018). For instance, inclusion of photosensitizers such as methylene blue (MB) and erythrosin B (ER) into PCL and PLGA electrospun membranes imparted visible light–dependent localized antimicrobial activity (Contreras et al., 2019). Photodynamic antimicrobial therapy is gaining considerable interests in the recent past. Very recently Carlos et al. reported the use of copper sulfide (CuS) nanoparticles together with indocyanine green (ICG) as a synergistic approach to effectively induce antimicrobial activity against pathogenic bacterial and fungal species (Garin et al., 2021). Near-infrared light irradiation triggered the generation of intra and extracellular species, thereby killing the microbial cells. It is also note that the combination is cytofriendly to mammalian cells prompting its use for biomedical applications. One may note that, such non-toxic combinations can be used into various electrospun nanofibers and impart antimicrobial activity. In synchrony with such cytofriendly routes, the methods reported by Jialong et al. are devoid of any toxic reducing agents or complex instruments for this process. These metallic nanoparticles can be applied as such or loaded into nanofibrous scaffolds to mediate its osteogenic functions. Homogeneity if achieved, it can fill into the nanofibers and may provide an excellent surface area. Lower the loading content will result in less impact on the biocompatibility. To achieve optimal action, sustained release should be ensured which requires extensive loading of nanoparticles. This will adversely affect the stability of the fibrous matrix, mechanical properties, and thermal conductivity leading to faster degradation of the PLLA matrix. In addition to this, the provision for loading pharmacological agents targeting bone would be hindered. Therefore, we realized that the approach by Jialong et al., is expected to facilitate 1) *in situ* nanoparticles formation on the PLLA fibers without the involvement of external reducing agents and 2) loading of drugs into the PLLA fibers (Figure 1). Also, the wettability and surface hydrophilicity of PLLA can be improved. Additionally, we also speculate researchers can extend this study for coating biomedical implants. Electrospinning can be carried out on metallic implants (used as a collector) using PLLA/other polymers and dip coating in PDA followed by immersion in respective metal precursor solution will aid in formation of metallic nanoparticles. PDA alone is reported to adhere to various substrates to covalently immobilize numerous biomolecules owing to its copious reactive functional groups (Singh et al., 2021). The polar groups present in PDA not only aids in the formation of nanoparticles but also will support attachment and growth of cells (Qiu et al., 2018). Therefore, versatility of PDA-based surface modification is of much interest in orthopedic implants.

There are some limitations in the present study. First, the size measurements of silver nanoparticles (AgNPs) formed on the surface is not reported. Silver nanoparticles are known to regulate cell functions and are size dependent. Silver nanoparticles at 50 nm were found to be cytotoxic compared to 3 µm silver nanoparticles. The concentrations to induce antibacterial effects greater than that cause cytotoxic effects (Albers et al., 2013). AgNPs with the average size of 18 nm was found to cause apoptosis and necrosis (Zielinska et al., 2016). AgNPs accumulations also lead to ROS generation in osteoclasts. Therefore, studies on size measurements of silver or other metal nanoparticles produced by this route are needed to assess the potential health risks. Alarming data confirms the threat of silver nanoparticles on living organisms. Silver nanoparticles can enter into the natural habitat and hierarchy of trophic chain. It is known to disrupt cellular processes in plants and aquatic organisms (Bundschuh et al., 2018). AgNPs can enter into the animal respiratory system and causes pulmonary inflammation. In rats, it is accumulated in the liver, lungs, and induced genotoxicity (Pulit-Prociak and Banach, 2016). Therefore, careful considerations are mandatory for using AgNPs in healthcare sectors. Next, the biocompatibility was performed by an indirect method using 48 h conditioned medium from PLLA@PDA membranes. This time point is not sufficient to predict the biocompatibility as it is dependent on 1) lactic and glycolic acids from PLLA and 2) silver nanoparticles form the PLLA surface. A longer time points must be considered to better predict the biocompatibility. Also, direct cell interaction studies on the construct will provide a better understanding on the toxicity. Therefore, when developing a PLLA construct coated with metallic nanoparticles mediated by PDA, one should consider the aforementioned factors in testing the biocompatibility.
